# Clinical value of PDCA circulation nursing in reducing nosocomial infection

**DOI:** 10.3389/fmed.2025.1656740

**Published:** 2026-01-02

**Authors:** Xia Wang, Li Gu, Wenying Chen, Chen Ma, Ran Li

**Affiliations:** Department of Infectious Diseases and Clinical Microbiology, Beijing Chao-Yang Hospital, Capital Medical University, Beijing, China

**Keywords:** PDCA cycle method, nosocomial infection, management quality, nursing, quality improvement

## Abstract

**Objective:**

To explore the effect of PDCA circulation nursing in nursing management of nosocomial infection.

**Methods:**

One hundred and twenty inpatients of our hospital from January 2019 to December 2021 were randomly selected as the subjects, and were divided into control group (*n* = 60 cases) and observation group (*n* = 60 cases) according to the time point. The control group implemented routine nursing management, and the observation group implemented PDCA cycle method. The nursing satisfaction, nosocomial infection rate, nursing work quality score, patient self-management ability, medical equipment and goods qualification rate and nurse professional skill score were compared between the two groups.

**Results:**

Compared with the control group, the observation group demonstrated significantly higher nursing quality scores (in medical device management, environmental management, cleaning and disinfection quality, and packaging quality), higher qualification rates (of package, sterilized articles, surgical instrument cleaning, and disinfectant concentration), and greater patient self-management ability (in self-management attitude, disease cognition, self-management skills, and complication prevention) (all *p* < 0.05). The overall nursing satisfaction in the observation group was 95.00%, significantly higher than 73.33% in the control group (*p* < 0.05). The incidence of nosocomial infection in the observation group was 5.00% (3/60), significantly lower than 16.67% (10/60) in the control group (*p* < 0.05). Additionally, nurses in the observation group scored higher on professional skills such as team spirit, communication and coordination, and problem-solving ability (all *p* < 0.05).

**Conclusion:**

The PDCA cycle method is effective in hospital infection management, significantly improving nursing quality, enhancing patient self-management and satisfaction, and reducing the incidence of nosocomial infection.

## 1 Introduction

Hospital-acquired infections, also defined as healthcare-associated infections (HAIs), represent a major global challenge to patient safety and healthcare systems. They refer to infections acquired by patients while receiving treatment for other conditions within healthcare facilities, which may occur during hospitalization or after discharge. These infections exclude any pre-existing infections or infections present at admission but not yet manifesting symptoms (1, 2). The hospital environment constitutes a unique system characterized by high pathogen concentration, continuous movement of patients and staff, and a concentration of immunocompromised individuals. The combined effects of diverse disease profiles, high personnel turnover, and susceptible hosts significantly increase the risk of cross-transmission and elevate the overall incidence of hospital-acquired infections (3). These infections lead to prolonged hospital stays, increased antimicrobial resistance, substantial additional healthcare costs, and heightened patient morbidity and mortality (4, 5).

To address this persistent threat, multiple strategies are currently employed to manage and prevent hospital-acquired infections. Traditional approaches typically include rigorous hand hygiene programs, enhanced environmental cleaning and disinfection, surveillance programs, and the implementation of isolation precautions (3, 6, 7). More recently, technological interventions such as automated hospital-acquired infection surveillance systems (RT-NISS), high-quality care initiatives, and advanced data analytics have been introduced (8, 9). While these measures are crucial, their effectiveness is often hindered by fragmented implementation, inadequate compliance, and a lack of continuous, systematic monitoring and feedback mechanisms. Many of these approaches function as isolated strategies rather than being integrated into a cohesive management system.

The PDCA cycle (Plan-Do-Check-Act) serves as the cornerstone of continuous quality improvement, providing a structured framework to overcome these limitations (10, 11). Numerous studies demonstrate the effective role of PDCA cycle management in the diagnosis and management of various diseases (12). For instance, the PDCA approach facilitated the development of optimized insulin infusion protocols and reduced the prevalence of hyperglycemia in critically ill patients (13). The PDCA cycle significantly impacted nutritional management for nasopharyngeal carcinoma (NPC) patients (14). It effectively enhanced healthcare providers’ adherence to sepsis bundle protocols, improving sepsis treatment efficiency (15). Compared to traditional techniques, the PDCA cycle embodies holistic and systematic principles. It does not seek to replace fundamental practices like hand hygiene but provides a robust operational framework to ensure these practices are effectively planned, reliably executed, rigorously reviewed, and continuously optimized (16, 17). At the same time, the PDCA cycle method constantly checks, finds and solves problems, formulates rectification countermeasures, clarifies the management direction in the next stage, takes the cycle evaluation results as the basis, so that the prevention and control of hospital infection has been in a benign cycle, and each link affects and restricts each other, which is conducive to the continuous optimization and smooth implementation of hospital infection prevention and control (18, 19).

Therefore, to rigorously evaluate its efficacy, this study employs a randomized controlled trial design to investigate the application of the PDCA cycle in hospital-acquired infection nursing management. The aim is to provide high-level evidence for its integration into standard infection control practices.

## 2 Materials and methods

### 2.1 Study design and subjects

This study is a randomized controlled trial with a total of 120 inpatients in our hospital. The study subjects were 120 subjects who were hospitalized between January 2019 and December 2021. A computer-generated random number sequence was used to assign patients to the observation group or the control group, with 60 cases in each group. The randomization sequence is hidden before the assignment is implemented. The control group consisted of 60 patients (31 males and 29 females) with an average age of (32.76 ± 4.23) years (23–41). Department Distribution: 12 obstetrics, 25 operating rooms, 9 medical wards, 4 intensive care units and 10 surgical wards. There were 60 cases in the observation group, 33 males and 27 females, with an average age of (33.54 ± 4.36) years (24–41). Department Distribution: 11 obstetrics, 23 operating rooms, 11 medical wards, 5 intensive care units and 10 surgical wards. There was no significant difference in clinical data between the two groups (*p* > 0.05).

This study protocol has been approved by the Ethics Committee of Beijing Chaoyang Hospital Affiliated to Capital Medical University. All participants had a full understanding of the purpose of the study and signed a written informed consent form before inclusion in the study.

### 2.2 Interventions

#### 2.2.1 Control group

Patients in the control group received routine nursing management for nosocomial infection. This included: (1) Adherence to standard hand hygiene protocols. (2) Performing routine environmental cleaning and disinfection. (3) Implementing isolation precautions based on clinical symptoms or physician orders. (4) Conducting general health education for patients.

#### 2.2.2 Observation group

In addition to routine nursing care, the observation group implemented the PDCA cycle management method. The specific procedures for each phase were as follows:

Plan (P)

(1) Problem analysis: The nursing management team first conducted a retrospective analysis of all nosocomial infection cases in the 6 months prior to the study. Data indicated that low hand hygiene compliance (<50%) and suboptimal cleaning of environmental surfaces were the primary contributing factors.
(2) Goal setting: Specific, measurable objectives were established: to increase hand hygiene compliance to >85% and to reduce the overall nosocomial infection rate by 25% within the study period.
(3) Action plan: A detailed “Patient-Centered Infection Control Work Plan” was formulated, which mandated regular training sessions for nursing staff (20).

Development (D)

(1) Structured training

Theoretical training: Weekly one-hour sessions were conducted, covering guidelines including the Diagnostic Standards for Nosocomial Infections and the *Technical Specifications for Disinfection of Medical Institutions (WS/T 367-2012). A closed-book post-training examination required a score of ≥80% to pass.

Operational training: Hands-on training and assessment were performed for key procedures such as “aseptic technique,” “intravenous infusion,” “ventilator circuit disassembly and disinfection,” and “medical waste classification.” The “see-one, do-one, teach-one” model was employed, and a score of ≥90% on a standardized checklist was required for competency (21).

(2) Enhanced protective measures

Hand hygiene: A hand hygiene compliance monitoring system was installed at the ICU entrance.

Environmental monitoring: Infection control nurses daily sampled high-touch surfaces in wards using ATP bioluminescence detectors. A relative light unit (RLU) value of <100 was set as the cleanliness benchmark.

High-risk patient management: Strict contact isolation was enforced for patients colonized or infected with multidrug-resistant organisms (MDROs). Patient and family education on infection prevention was intensified (22).

Check (C)

(1) Process supervision: Designated infection control professionals conducted unannounced, on-site inspections at least twice weekly to audit compliance with the new protocols.
(2) Outcome assessment: The results of the ATP surface monitoring and hand hygiene compliance data were collected and analyzed weekly.
(3) Performance management: Audit results were incorporated into the nurses’ monthly performance evaluations, linked to a clear reward and penalty system to ensure accountability.

(1) Assessment (A)

(1) Monthly review meetings: The infection control team held monthly meetings to analyze inspection data, identify persistent problems (e.g., inconsistent disinfectant concentration preparation), and determine root causes.
(2) Continuous improvement: Based on this analysis, corrective actions were formulated (e.g., re-training on disinfectant dilution procedures). These measures were then fed back into the next PDCA cycle, initiating a new round of Plan phase for further refinement.

### 2.3 Observation index

(1) Infection rate: Record and count the nosocomial infection rate before and after the implementation of PDCA. According to the Diagnostic Criteria for Nosocomial Infection (Trial) promulgated by the Ministry of Health of China, nosocomial infection cases occurring during hospitalization in both groups of patients were diagnosed and recorded. Nosocomial infection rate (%) = (number of new nosocomial infections in the same period/total number of hospitalized patients during the observation period) × 100%.
(2) Score of professional skills of nurses: Assessed using a validated self-developed scale measuring team spirit, communication, responsibility, and problem-solving. Each of the five domains was scored out of 20. The scale demonstrated high reliability in a pre-test (Cronbach’s *α* = 0.92). Assessments were performed by head nurses and department directors blinded to group allocation.
(3) Scores of nursing work quality: We use the Nursing Quality Checklist formulated with reference to national standards such as the “Management Code for Hospital Disinfection Supply Centers (WS 310)” and the “Management Code for Cleaning and Disinfection of Environmental Surfaces in Medical Institutions (WS/T 512).” The list covers medical device management, environmental management, cleaning/disinfection quality, and packaging quality, with a full score of 100 points for each item. The assessment is carried out by the Quality Control Specialist of the Nursing Department who is not involved in the grouping.
(4) Nursing satisfaction: 80 nursing satisfaction questionnaires were delivered to each department before and after PDCA cycle was applied. The nursing satisfaction questionnaire is self-made by the hospital. The score includes 5 items, with a full score of 100, including very satisfied (85–100), satisfied (70–84) and dissatisfied (<69). The satisfaction of each department = very satisfied rate + satisfied rate.
(5) Patient self-management ability: The Self-Management Ability Scale for Patients with Chronic Diseases was used to measure the sinicization and reliability validity, and the Cronbach’s *α* coefficient was 0.85 in the study population. The patient’s self-management ability mainly includes self-management attitude, disease cognition, self-management ability and complication prevention. The score of these four items is 0–5. The higher the score, the stronger the patient’s self-management ability.
(6) Qualification rate of medical devices and articles: The qualified rates of medical devices and articles in the two groups were compared, including: the qualified rate of packaging, the qualified rate of sterilization articles, the qualified rate of cleaning surgical instruments, and the qualified rate of disinfectant concentration. Professionals from the hospital infection control department will conduct sampling inspections according to the following national standards to calculate the pass rate. Packaging pass rate: Complies with the provisions of WS 310.2 on device packaging, sealing and labeling. Passage rate of sterile items: The appearance of the sterile package is not damaged or damp, and the chemical indicators are discolored up to standard, within the validity period. Qualified rate of surgical instrument cleaning: ATP biofluorescence detection method is used, and the relative light unit value (RLU) ≤200 is qualified. Disinfectant concentration pass rate: Use a concentration test card for testing, and the concentration is qualified within the standard use range.

### 2.4 Statistical analysis

SPSS 20.0 software was used to analyze and process the obtained data. The measurement data were expressed in (x ± s) and *t*-test was used. The counting data is expressed as a percentage (%) *χ*^2^ inspection. With *p* < 0.05 as the difference with statistical significance.

## 3 Results

### 3.1 Comparison of nosocomial infection rates between two groups

**Figure 1:**
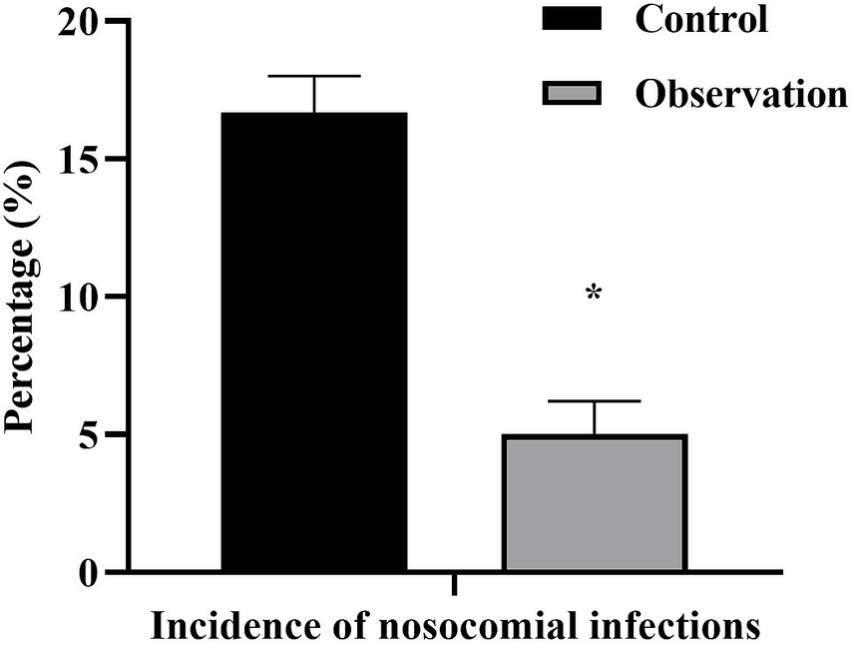
Comparison of nursing quality scores between two groups. ^*^p < 0.05, compared with the control group.

After PDCA nursing was applied, the incidence of nosocomial infection in the observation group (5%) was significantly lower than that in the control group (16.67%) (𝜒^2^ = 4.627, *p* = 0.031). As shown in Figure 1.

### 3.2 Comparison of professional skills between two groups of nurses

**Figure 2:**
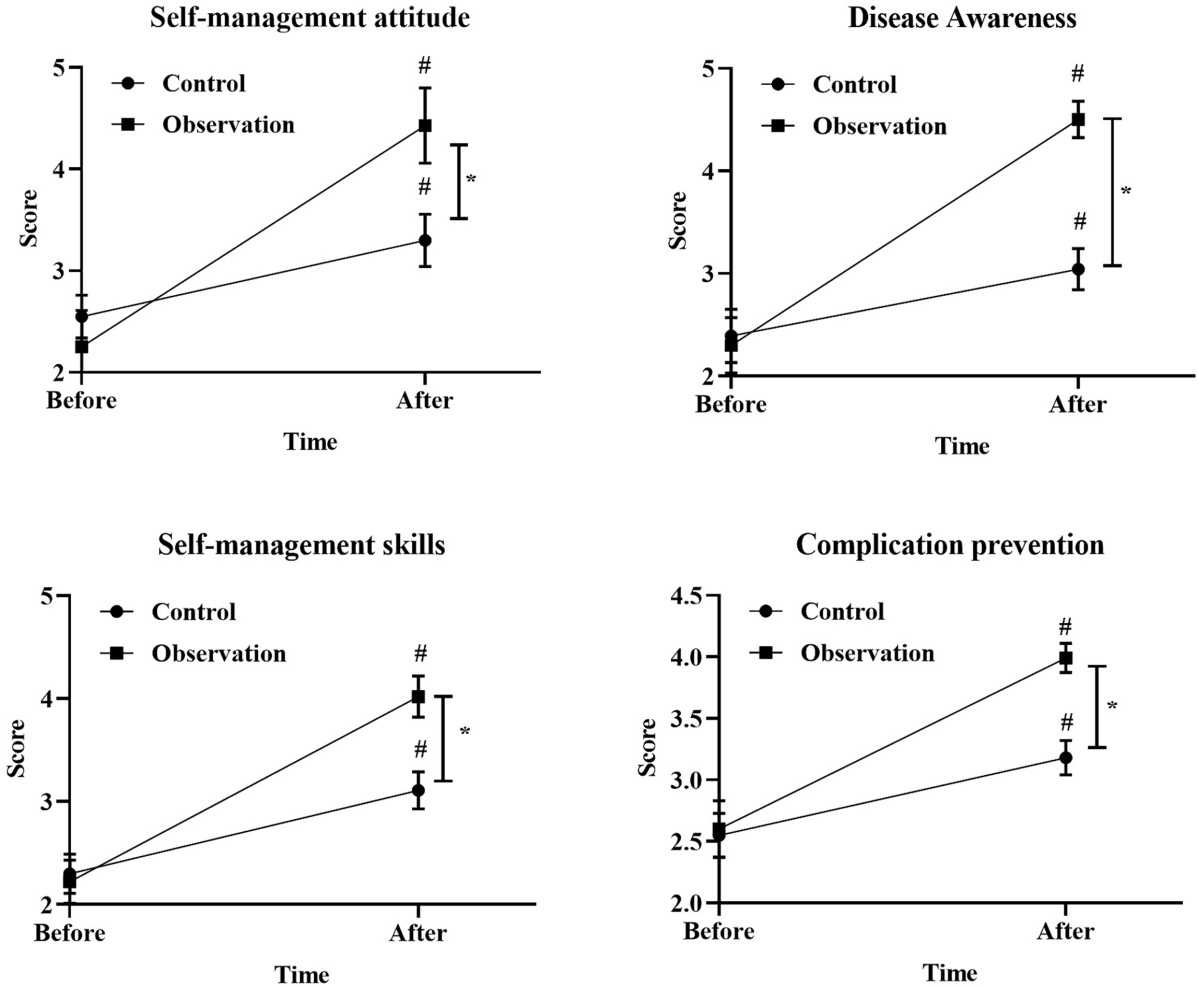
Comparison of nosocomial infection rates between two groups. ^*^p < 0.05, compared with the control group.

After the PDCA cycle was implemented, the scores of self-management attitude, disease awareness, self-management skills and complication prevention in the observation group were significantly higher than those in the control group (*p* < 0.05), as shown in Figure 2.

### 3.3 Comparison of nursing quality scores between two groups

**Figure 3:**
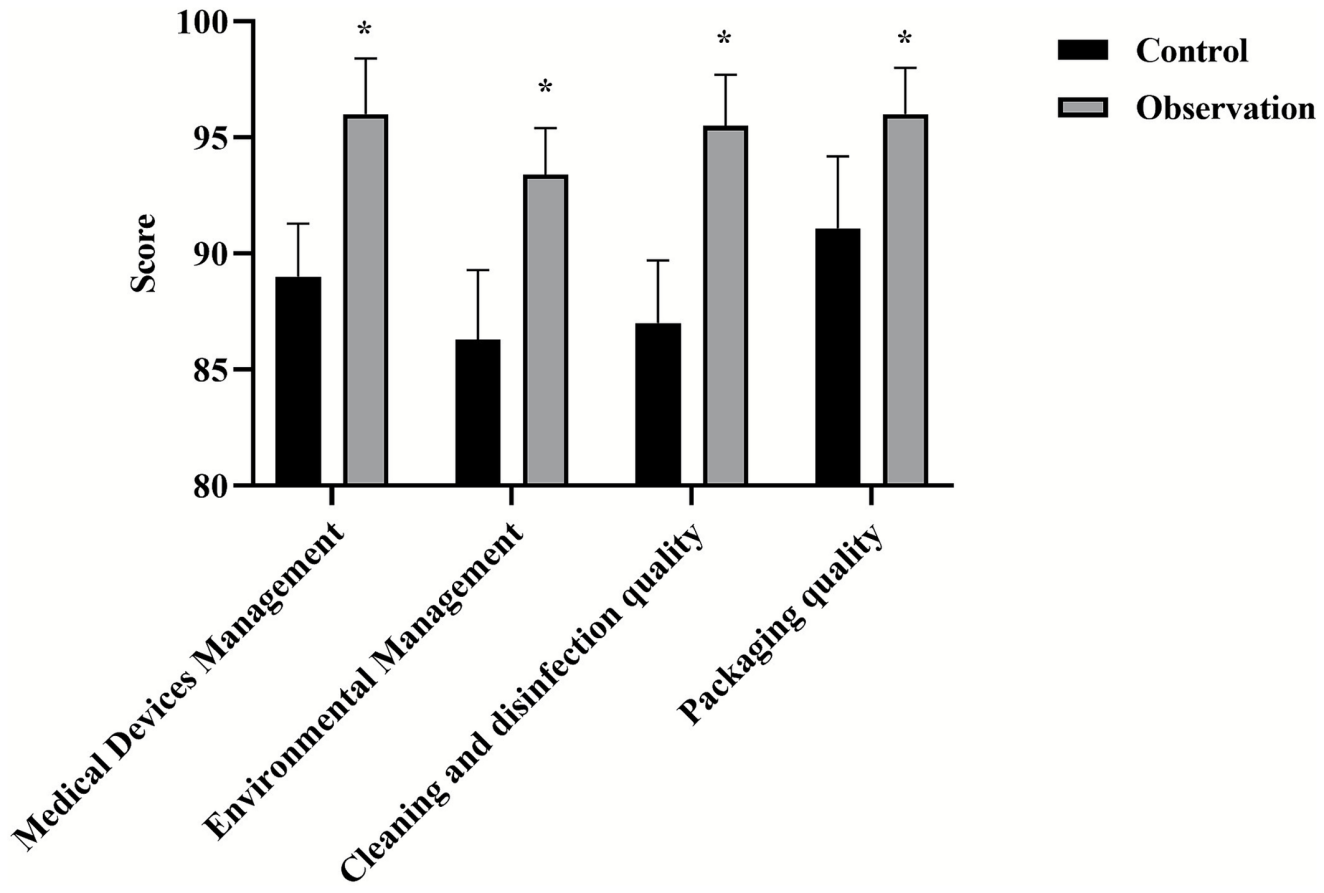
Comparison of professional skills between two groups of nurses. ^*^p < 0.05, compared with the control group, ^#^*p* < 0.05, compared with before nursing.

All scores of nursing quality (medical device management, environmental management, cleaning and disinfection quality, packaging quality) in the observation group were higher than those in the control group (*p* < 0.05), as shown in Figure 3.

### 3.4 Comparison of department satisfaction between two groups

The total satisfaction of each department in the observation group (95%) was significantly higher than that in the control group (73.33%) (𝜒^2^ = 11.346, *p* < 0.01). As shown in Table 1.

**Table 1:** Comparison of department satisfaction between two groups.

Groups	*N*	Very satisfied	Satisfied	Dissatisfied	Total satisfaction
Observation group	60	31 (51.67%)	26 (43.33%)	3 (5%)	95%
Control group	60	20 (33.33%)	24 (40%)	16 (26.67%)	73.33%
𝜒^2^		—	—	—	11.346
*p*		—	—	—	<0.01

### 3.5 Comparison of self-management ability scores between two groups

The self-management attitude, disease awareness, self-management skills and complication prevention score of patients in the observation group were significantly higher than those in the control group after nursing (*p* < 0.05). As shown in Table 2.

**Table 2:** Comparison of self-management ability scores between two groups.

Groups	*N*	Self management attitude		Disease awareness		Self management skills		Complication prevention	
		Before	After	Before	After	Before	After	Before	After
Observation group	60	2.25 ± 0.23	4.01 ± 0.34	2.77 ± 0.27	4.13 ± 0.21	2.83 ± 0.43	4.32 ± 0.33	2.34 ± 0.38	4.12 ± 0.50
Control group	60	2.32 ± 0.44	3.51 ± 0.46	2.81 ± 0.17	2.92 ± 0.21	2.76 ± 0.23	3.21 ± 0.47	2.44 ± 0.470	3.02 ± 0.33
*t*		1.092	6.771	0.9711	9.271	1.112	14.97	1.282	14.22
*p*		0.277	<0.05	0.3335	<0.05	0.2684	<0.05	0.2025	<0.05

x ± s = mean ± standard deviation.

### 3.6 Comparison of qualified rate of medical devices and articles between two groups

The qualified rate of package, sterilized articles, surgical instrument cleaning and disinfectant concentration in the observation group were higher than those in the control group (p < 0.05), as shown in Table 3.

**Table 3:** Comparison of qualified rate of medical devices and articles between two groups.

Groups	*N*	Packaging qualified	Sterilized articles are qualified	Cleaning of surgical instruments is qualified	The concentration of disinfectant is acceptable
Observation group	60	61 (95.65)	62 (96.44)	61 (96.55)	62 (98.44)
Control group	60	51 (84.35)	52 (85.68)	50 (83.36)	53 (88.35)
𝜒^2^		5.254	4.544	5.523	4.633
*p*		<0.05	<0.05	<0.05	<0.05

## 4 Discussion

**Figure 4:**
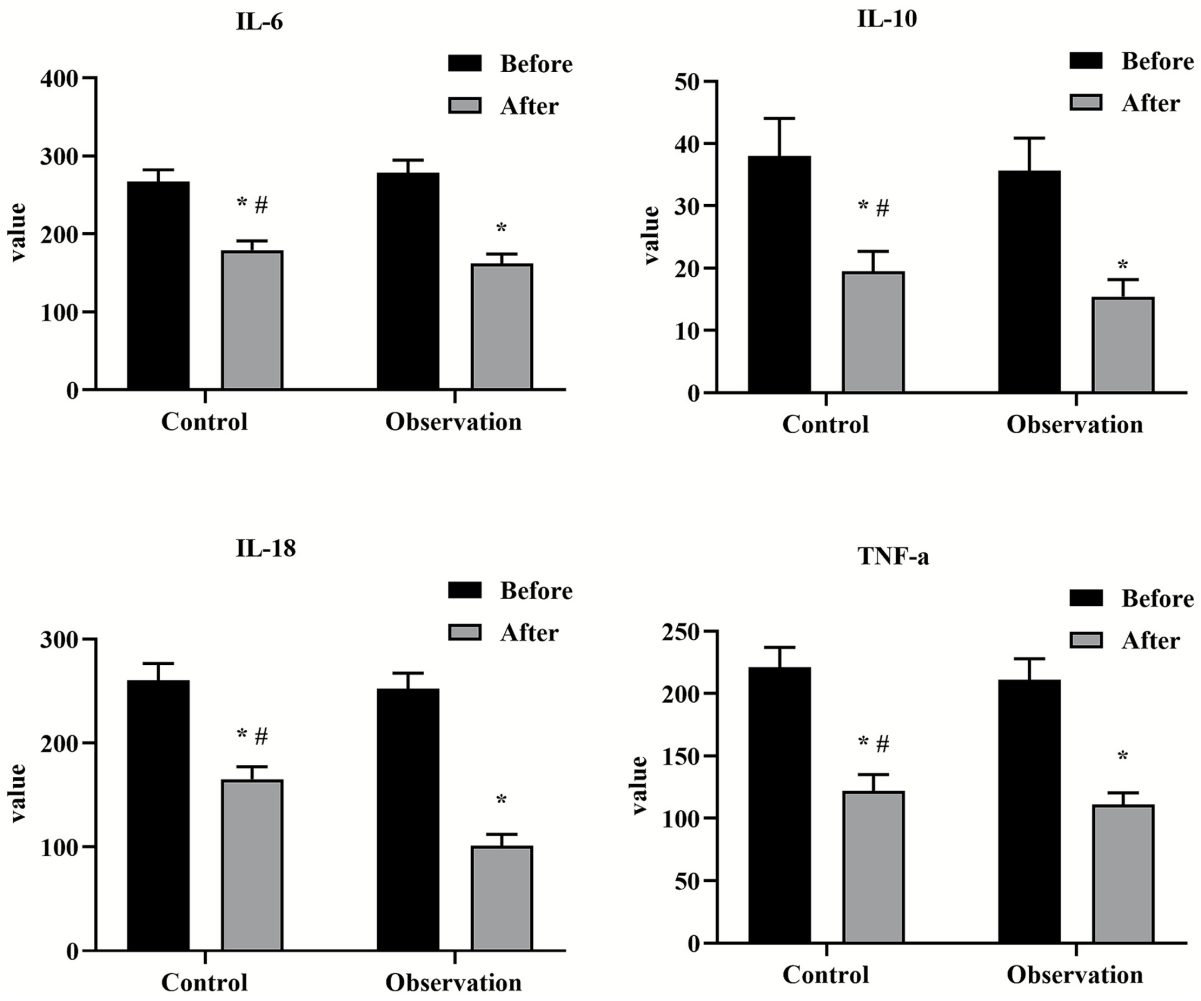
Comparison of qualified rate of medical devices and articles between two groups. ^*^p < 0.05, compared with the control group.

**Figure 5:**
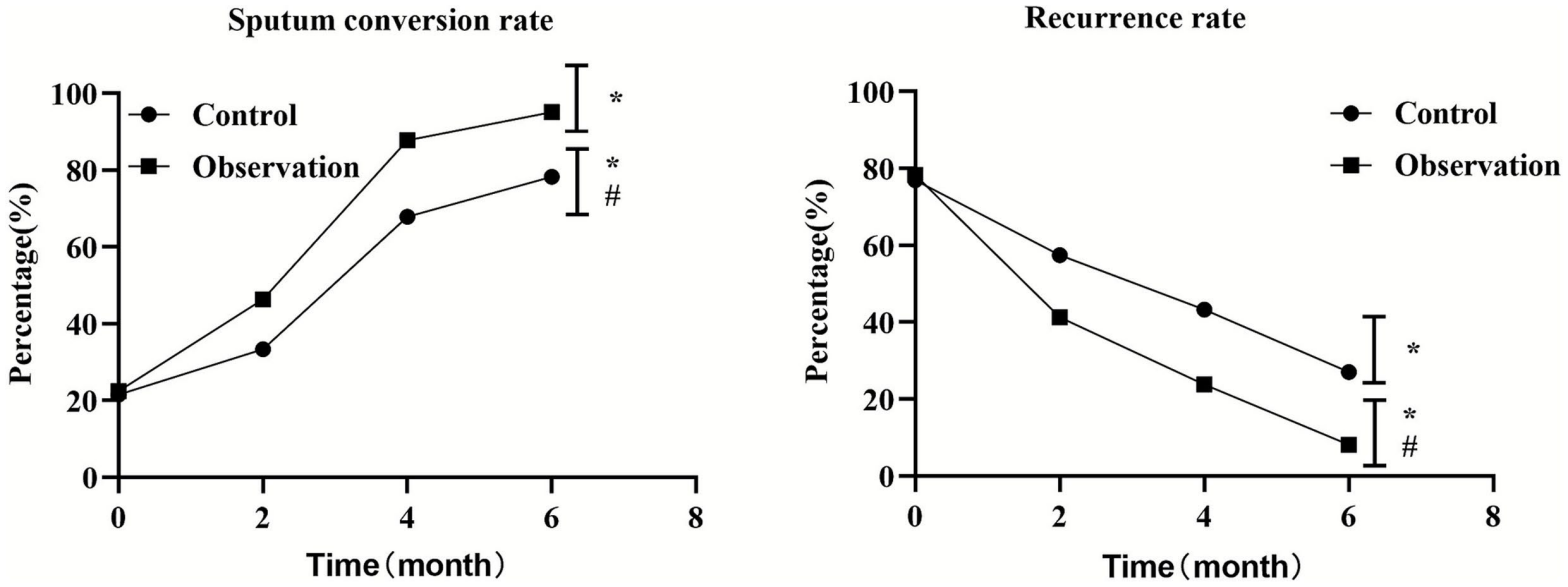
Comparison of self-management ability scores between two groups before and after nursing. ^*^p < 0.05, compared with the control group, ^#^*p* < 0.05, compared with before nursing.

This study systematically evaluated the effect of PDCA cycle in the management of nosocomial infection care through a randomized controlled trial. The results consistently showed that the implementation of PDCA cycle could significantly improve nursing quality, nurse professional skills, medical device qualification rate and patient satisfaction, and effectively reduce the nosocomial infection rate compared with conventional management. This section will provide an in-depth look around these key findings (see Figures 4, 5).

### 4.1 The role of PDCA cycle on nurses’ professional skills and nursing quality

The results of this study showed that the nurses in the observation group were significantly better than the control group in terms of professional skills such as team spirit, communication and coordination, and problem-solving ability. This finding echoes the results of previous studies (18). The inherent reason may be that the PDCA cycle is not a passive management instruction, but an active, participatory management tool. In the “planning” stage, nurses participate in problem analysis, enhancing their sense of ownership and analytical skills. In the “check” and “process” phases, regular feedback is associated with performance to give clear goals and motivation for improvement. This continuous learning and improvement environment naturally promotes the improvement of nurses’ comprehensive abilities. At the same time, the standardized checklist based on national specifications (such as WS 310, WS/T 512) has shifted the nursing quality assessment (medical device management, environmental cleaning, etc.) from subjective judgment to objective quantification, ensuring the consistency of the PDCA cycle in improving work quality.

### 4.2 Effectiveness in disinfection and sterilization pass rate and infection control

This study found that the pass rate of the observation group in key indicators such as device packaging, cleaning quality and disinfectant concentration was significantly improved, which directly led to a decrease in the nosocomial infection rate from 16.67% in the control group to 5.00% in the observation group. Previous studies have shown that the application of PDCA nursing mode can effectively improve the qualified rate of cleaning and disinfection, rust removal, and disinfectant concentration of surgical instruments, which is similar to the results of this study (19, 23). The core advantage of the PDCA cycle is its ability to establish a “data-driven” closed loop of continuous improvement. In the “treatment” stage, the root cause analysis and retraining of the non-conformities are carried out in a targeted manner, so as to solve the problem from the source. This evidence-based, preventive management model is far more effective and economical than the traditional model of dealing with infection after it occurs.

### 4.3 Impact on patients’ self-management ability and satisfaction

It is important to note that the benefits of the PDCA cycle are not limited to caregivers and hardware processes, but also extend to the patient level. In this study, the satisfaction of patients in all departments increased from 73.33 to 95%, which is consistent with the published results (24). The scores of patients in the observation group were significantly higher in self-management attitude and disease cognition. This may be due to the inclusion of “enhanced patient education and advocacy” as a key measure in the PDCA framework. When nurses receive more systematic training, the health education they provide to patients is also more targeted and effective, thereby empowering patients and improving their self-management skills. When patients feel a cleaner environment, more standardized operations, more professional communication, and a lower risk of infection, their overall satisfaction naturally increases.

### 4.4 Limitations and prospects

Despite the positive results of this study, there are some limitations. First, this is a single-center study with a relatively limited sample size, and the rigor of the results needs to be further verified by multicenter, large-sample studies. Second, the long-term sustainability of the intervention effect needs to be observed at a longer follow-up. In addition, the successful implementation of the PDCA cycle is highly dependent on the support of hospital management and collaboration between departments, which may face different challenges in different medical institutions.

## 5 Conclusion

In summary, this study shows that PDCA cycle, as a structured quality management method, can effectively integrate and optimize the nursing management process of nosocomial infection. It achieves the comprehensive goal of enhancing patient safety and satisfaction by improving the professional ability of nurses, realizing the standardization and quantitative management of nursing quality, and ensuring continuous disinfection and sterilization pass rates. Therefore, PDCA cycle is a valuable and generalizable management strategy in the field of nosocomial infection control.

## Data Availability

The datasets presented in this study can be found in online repositories. The names of the repository/repositories and accession number(s) can be found in the article/supplementary material.
